# Comparative analysis of *Bacillus pumilus* TUAT1 endospores and vegetative cells: Implications for plant growth promotion and soil microbiome modulation

**DOI:** 10.5511/plantbiotechnology.25.0328a

**Published:** 2025-09-25

**Authors:** Shin-ichiro Agake, Jean Louise Cocson Damo, Hiroki Rai, Gary Stacey, Michiko Yasuda, Naoko Ohkama-Ohtsu

**Affiliations:** 1 Institute of Global Innovation Research, Tokyo University of Agriculture and Technology, 3-8-1 Harumicho, Fuchu, Tokyo 183-8538, Japan; 2 Divisions of Plant Science and Technology, University of Missouri, 1201 Rollins St., Columbia, MO 65201-4231, USA; 3 National Institute of Molecular Biology and Biotechnology, University of the Philippines Los Baños, Los Baños, Laguna 4031, Philippines; 4 Department of Biological Production, Faculty of Bioresource Sciences, Akita Prefectural University, 241-438 Kaidobata-Nishi, Shimoshinjo-Nakano, Akita-shi, Akita 010-0195, Japan; 5 Institute of Agriculture, Tokyo University of Agriculture and Technology, 3-8-1 Harumicho, Fuchu, Tokyo 183-8538, Japan

**Keywords:** *Bacillus pumilus*, endospores, microbiome analysis, PGPR, *Setaria viridis*

## Abstract

*Bacillus pumilus* TUAT1, a gram-positive and spore-forming plant growth-promoting rhizobacterium, has been utilized as a biofertilizer due to its robust ability as spores to withstand environmental stresses and ensure long-term viability. This study investigated the mechanisms underlying the plant growth-promoting effects of spores and vegetative cells. Elemental analyses revealed that endospores are enriched in carbon, calcium, and manganese, which contribute to their protective properties, while vegetative cells are richer in nitrogen and phosphorus. Notably, both viable and dead spores and vegetative cells promoted the growth of *Setaria viridis* in natural soil. Microbial community analysis showed that bacterial alpha diversity was not changed across treatments, whereas beta diversity varied significantly, forming distinctly separated groups influenced by planting and inoculation. Fungal community analysis exhibited increased alpha diversity due to *Setaria* planting but no significant effects from bacterial treatments. Enrichment of *Bdellovibrio* spp., *Bacteriovorax* spp., and *Pseudomonas* spp. in soil inoculated with viable and dead vegetative cells and spores highlighted the capability of indirect mechanisms through microbial interactions rather than direct nutrient supply from bacterial residues. We believe that bacterial inoculants, including dead cells, modulate soil microbial communities to enhance plant growth, emphasizing their potential in sustainable agriculture.

## Introduction

The endospores of *Bacillus pumilus* TUAT1, a gram-positive and spore-forming bacterium isolated as an endophytic PGPR, have been used as an inoculant in the commercial biofertilizer, named “Yume-bio” (Agake et al. 2022a). Bacterial spores are physiologically dormant and resistant to environmental stresses, such as extreme temperatures, ultraviolet radiation, and chemicals. Hence, the use of spores in “Yume-bio” allows for long-term storage and viability as a biofertilizer. Furthermore, we reported that *B. pumilus* TUAT1 inoculation using spores induced stronger plant growth promotion than inoculation with vegetative cells and even autoclaved dead spores promoted plant growth of *Oryza sativa* and *Setaria viridis* (Agake et al. 2022b; Ngo et al. 2019; Seerat et al. 2019). The components of bacterial spores differ from those of vegetative cells and, therefore, the question of what nutrients from spores may influence plant growth promotion remains.

Developments and improvements in high-throughput next-generation sequencing have revealed that the microbiome in soil and rhizosphere plays a crucial role in plant growth promotion and the biofertilizer application modules microbial diversity of rhizosphere (Wang et al. 2021; Zhang et al. 2021; Zhou et al. 2024). Previously, we reported the effects of biofertilization using “Yume-bio” on the microbial community, with forage rice as the plant material, focusing on observed changes in alpha and beta diversities (Win et al. 2020). However, the relationship between plant growth promotion due to vegetative cells and spores and the microbiome is still unclear. Hence, in this work, we directly assayed the effect of vegetative and spore inoculation on the soil microbiome of the C4 model plant, *Setaria viridis.*

## Materials and methods

### Bacterial inoculant preparation

The preparation of bacterial materials was described previously (Agake et al. 2022b). Briefly, vegetative cells and endospores were cultured in tryptic soy broth (TSB) and Difco sporulation medium (DSM), respectively (Nicholson and Setlow 1990). One milliliter of preculture was transferred into 300 ml of TSB and incubated with shaking overnight resulting in a large population of vegetative cells, as confirmed by phase contrast microscopic observation (>99.9%). The endospore solution was prepared by culturing cells in DSM for 3 days with purity again confirmed by microscopy (>99.9%). Both vegetative cells and spores were concentrated by centrifugation and washed 3 times and resuspended with 0.85% saline water. The resuspended vegetative cells and spores were diluted 100-fold, resulting in 1×10^7^ CFU ml^−1^ for viable inoculants or autoclaved at 121°C for 40 min for residue applications.

### Elemental analyses of vegetative cells and spores

Approximately 20 mg of lyophilized bacterial pellets (i.e., vegetative cells and endospores) were analyzed for total nitrogen and carbon using a NC analyzer SUMIGRAPH NC TR-22 (Sumika Chemical Analysis Service Ltd., Tokyo, Japan). The analysis was performed with 5 replicates. For total phosphorus measurement and ionome analysis, 80 mg of the bacterial cells were digested by 2 ml of HNO_3_ and 0.2 ml of H_2_SO_4_ at 150°C (Kuboi 1990). The digested solutions were diluted with 0.08 N HNO_3_ up to 15 ml. The colorimetric method for total phosphorus was conducted using MoO_12_S_3_ with 3 replicates (Murphy and Riley 1962). Ionome analysis of digested vegetative cells and spores was conducted using an inductively coupled plasma optical emission spectrometer (ICP-OES, iCAP 6000 SERIES, Thermo Fisher Scientific Inc., Waltham, MA, USA).

### Plant growth assay and collecting soil samples

*Setaria viridis* A10.1 was used as a plant material. Seed sterilization and germination processes were described previously (Agake et al. 2022b). In brief, the thick seed coats were scarified by 95% (v/v) sulfuric acid for 15 min, followed by washing with sterile water. The seeds were further treated with 1% (v/v) sodium hypochlorite solution containing 0.1% (v/v) Tween 20 for 3 min and then washed 3 times. The sterilized seeds were sown on the half concentration of Murashige and Skoog (MS) agar plates (Murashige and Skoog 1962). The MS plates were incubated at 30°C overnight to enhance germination. The germinated plants on MS plates were moved to the growth chamber controlled as 16 h light (250 µmol s^−1^ m^−2^) at 28°C/8 h dark at 25°C for 4 days. Each germinated seedling was transplanted into a 50 ml falcon tube containing 40 ml of Andosol soil collected from the field located at the Tokyo University of Agriculture and Technology (35°41′03.4″N 139°29′00.7″E), and 20 plants were prepared for each treatment. Prior to use, the soil was filtered through a 1 cm sieve to remove litter and stones. An aliquot of soil was collected and frozen at −80°C to investigate the native microbiome. Each transferred plant was inoculated by applying 1 ml of the designated treatments, i.e., 0.85% saline solution, autoclaved dead vegetative cells (ADV), viable vegetative cells, autoclaved dead spores (ADS), or viable spores, directly to the soil at the base of the plant immediately after transplantation. The plants were then cultivated for 14 days in the growth chamber with the same conditions above. The soil samples without a plant were also treated with saline solution and incubated at the same condition to check the microbiome variability. The plants were irrigated with sterilized water and maintained 60% moisture until harvest. The plants were harvested 14 days after transplanting and the growth parameters were assessed while the soils were collected for further analysis.

### Soil microbial DNA extraction, library preparation, and amplicon analysis

Soil DNA extraction was performed in triplicate using the ISOIL kit for bead beating (NIPPON Genetics Co., Ltd., Tokyo, Japan) according to the manufacturer’s protocol. For each extraction, 0.5 g of soil was collected from the area surrounding the roots after carefully removing the plant from the tube. While this soil is located near the roots, it is not strictly rhizosphere soil as defined by root-adhering soil. Each soil sample was transferred into the 1.5 ml tubes for DNA extraction. Bacterial and fungal cells in the soil were disrupted through bead beating, and DNA was extracted using chloroform. The extracted DNA was washed with buffer and 70% (v/v) ethanol, then dissolved in 100 µl of TE buffer (pH 8.0). DNA concentrations were measured by NanoDrop 1000 (Thermo Fisher Scientific) and subsequently stored at −30°C until use.

The library preparations for 16S V3/V4 and ITS KYO1/KYO2 regions were performed by 2-step tailed PCR following the instructions of the Bioengineering Lab. Co., Ltd. (Kanagawa, Japan) as we reported previously (Damo et al. 2023). In brief, the 1st PCR amplification for V3/V4 and KYO1/KYO2 were conducted with 1st_PCR_V3V4f_MIX and 1st_PCR_V3V4r_MIX primer set or 1st_ITS1-F_KYO1 and 1st_ITS2_KYO2 using KOD-Plus-Neo enzyme (Toyobo Co., Ltd., Osaka, Japan) (Supplementary Table S1). The PCR products were purified using the FastGene Gel/PCR Extraction Kit (NIPPON Genetics Co., Ltd., Tokyo, Japan) and then used as templates for the tailed PCR using the 2nd Forward primer and 2nd Reverse primer set (Supplementary Table S1). Amplified PCR products were checked by electrophoresis and purified using the FastGene Kit. The PCR products were quantified using the Synergy H1 microplate reader (BioTek, Winooski, VT, USA) in combination with the QuantiFluor dsDNA system. Library quality was assessed with a Fragment Analyzer and the dsDNA 915 reagent kit (Advanced Analytical Technologies, Inc., Ankeny, IA, USA). Sequencing was conducted on the MiSeq platform using the MiSeq Reagent Kit v3.0 (Illumina, San Diego, CA, USA). The raw sequencing data obtained were subsequently analyzed using the Quantitative Insights into Microbial Ecology software (QIIME2.0). The sequenced data were further analyzed in the MicrobiomeAnalyst website tool (Chong et al. 2020; Dhariwal et al. 2017; Lu et al. 2023).

### Statistical analysis

All data were statistically analyzed using Microsoft Excel 365 (Microsoft Corporation, Redmond, WA, USA) for Student’s *t*-test, and SPSS Statistics version 29 (IBM SPSS Statistics, Armonk, NY, USA) for one-way ANOVA with post-hoc Tukey’s or Dunnett’s tests.

## Results and discussion

### The component differences between vegetative cells and endospores

**Figure 1:**
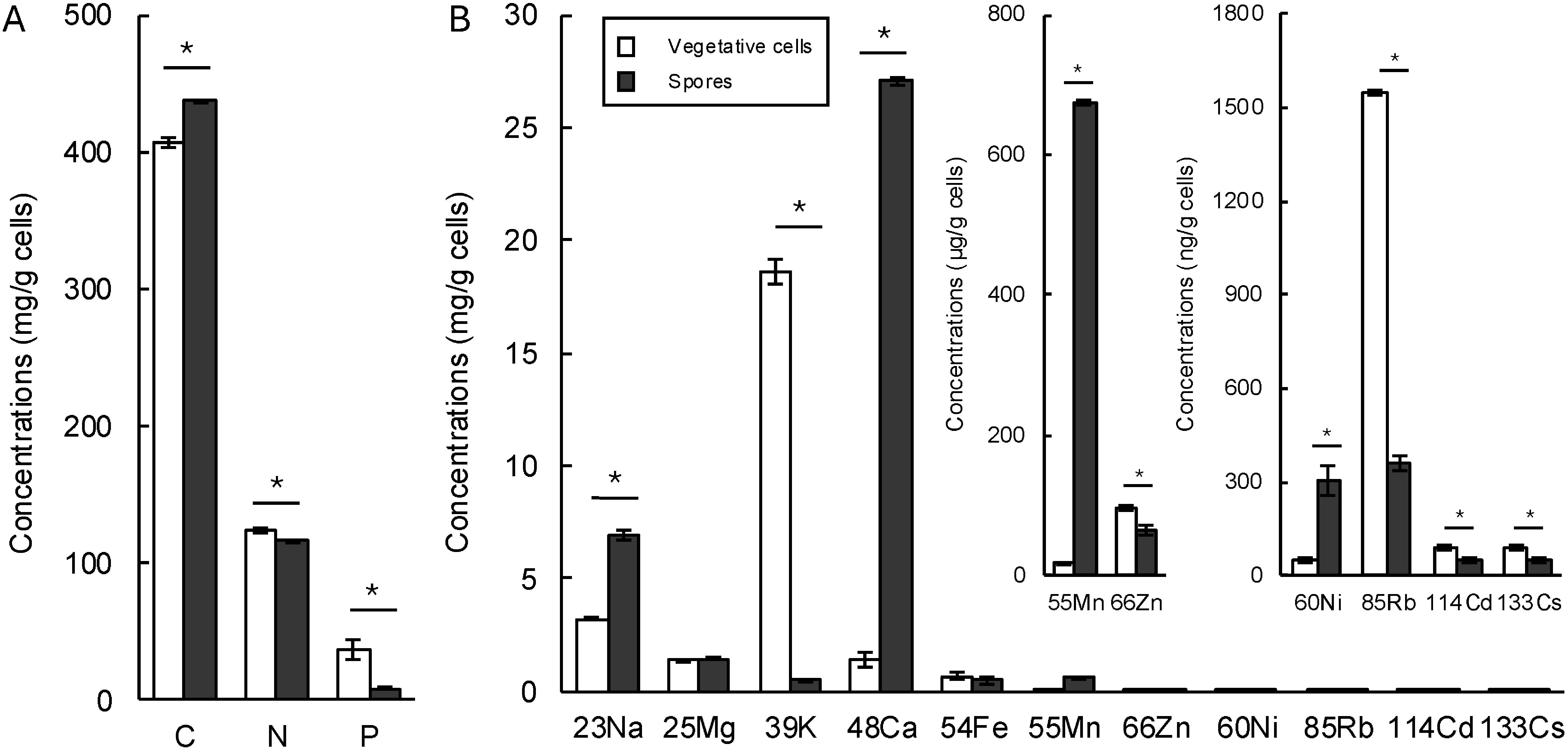
Figure 1. Element analysis results of vegetative cells and spores of *Bacillus pumilus* TUAT1. A, Carbon and nitrogen were analyzed using a NC analyzer, while phosphorus was determined via a colorimetric method employing molybdenum sulfate. B, Ionome analysis results obtained through ICP-OES. The NC analyzer results are based on five replicates, while the colorimetric analysis and ICP-OES results are based on three replicates each. An asterisk indicates significance, calculated using Student’s *t*-test with a 5% probability threshold.

The results for elemental analyses of vegetative cells and spores using the NC analyzer for nitrogen and carbon, the colorimetric method with molybdenum sulfate for phosphorus, and ICR-OES for other elements are shown in Figure 1. Among 14 elements, 12 elements showed significant differences between vegetative cells and spores, except for magnesium and iron. Spores contained more carbon, sodium, calcium, manganese, and nickel while vegetative cells were comprised of more nitrogen, phosphorus, potassium, zinc, rubidium, cadmium, and cesium.

Calcium, a well-known element enriched in spores, binds to dipicolinic acid (DPA) contributing to several characteristics such as protecting chromosome DNA from ultraviolet lights and stabilizing spore coats (Magge et al. 2008; Setlow et al. 2006; Slieman and Nicholson 2001). Similarly, manganese was shown to bind with DPA, which protects restriction enzymes from ionizing radiation (Granger et al. 2011). A higher amount of carbon in spores also reflects the thick polysaccharide spore coat and cortex (McKenney et al. 2013; Riley et al. 2021). However, the data did not show a significant enrichment in nutrients (e.g., N and P) on spores that might affect plant growth promotion. Vegetative cells may contain higher levels of amino acids and proteins, which contribute to nitrogen abundance, as well as ATP, which reflects phosphorus content, due to their higher metabolic activity compared to spores.

### The plant growth promotion by autoclaved dead cells

**Figure 2:**
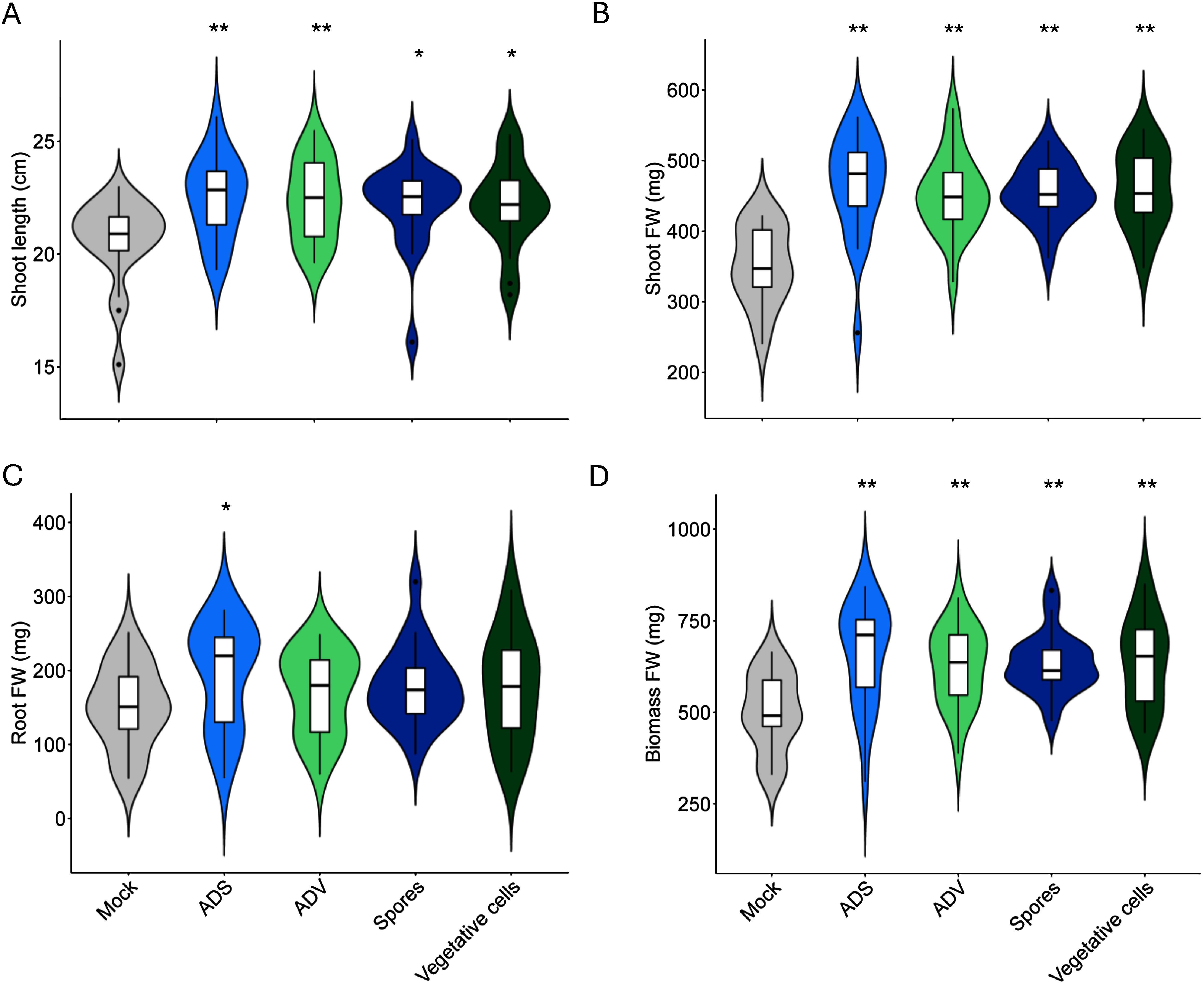
Figure 2. Plant growth parameters treated with bacteria and their autoclaved residues. A, Shoot length. B, Shoot fresh weight. C, Root fresh weight. D, Biomass fresh weight. Mock, saline solution; ADS, autoclaved dead spores; ADV, autoclaved dead vegetative cells; Spores, 1×10^7^ CFU ml^−1^ of *B. pumilus* TUAT1 spores; Vegetative cells, 1×10^7^ CFU ml^−1^ of *B. pumilus* TUAT1 vegetative cells. The error bar indicates a standard error, and an asterisk indicates significance calculated using Dunnett’s test with a 5% probability threshold (*n*=20).

We decided to conduct the plant assay with ADV, ADS, viable vegetative cells, and viable spores using plants grown in field soil. The various inoculants were resuspended in 0.85% saline solution with uninoculated saline serving as the mock treatment. Previously, we reported that 1×10^9^ CFU/ml of ADS stimulated plant while 1×10^7^ CFU/ml of viable spores showed the strongest and most stable effects. Interestingly, all treatments excepting mock promoted plant growth attributable to shoot length, shoot fresh weight, and biomass (Figure 2A, B, D and Supplementary Figure S1). Root growth, which is a key phenotype affected by B. *pumilus* TUAT1 spore inoculation, was enhanced at the ADS treatment (Figure 2C). However, ADV did not improve the root growth even though nutrients such as N, P and K were enriched in vegetative cells’ components (Figure 1). Those results supported our hypothesis that the plant growth-promoting effects of spores were mediated by mechanisms that include the improvement of nutrient uptake from the soil beyond direct nutrient supplementation. On the other hand, we did not observe the improvement of root growth by variable spore and vegetative cell inoculations while they promoted shoot growth. The effects of *B. pumilus* TUAT1 strain on the roots at nursery stages were previously characterized using sterilized soils (Agake et al. 2022a, b; Ngo et al. 2019). Hence, there is the possibility that *B. pumilus* TUAT1 may influence plant growth through indirect effects on the soil and rhizosphere microbiome.

### Response of the microbiome to *Setaria* planting and bacterial inoculation

Regarding the soil bacterial microbiome, 550,027 total rRNA sequence read counts were observed with an average of 26,191 sequences (22,524 as minimum and 44,823 as maximum). Total reads were clustered into 4,463 microbial amplicon sequence variants (ASV). In the soil fungal microbiome, 966,379 total ITS sequence read counts were obtained with an average of 46,018 sequences (36,867 in the minimum and 63,268 in the maximum). Total reads converged into 16,00 fungal ASV. ﻿The rarefaction curves were saturated at approximately 7,000 sequences in the bacterial analysis and 12,000 sequences in the fungal analysis (Supplementary Figure S2). Rarefaction curves of ITS samples indicated a trend of lower observed richness in the soil samples before incubation, while no differences were observed among treatments on rRNA.

**Figure 3:**
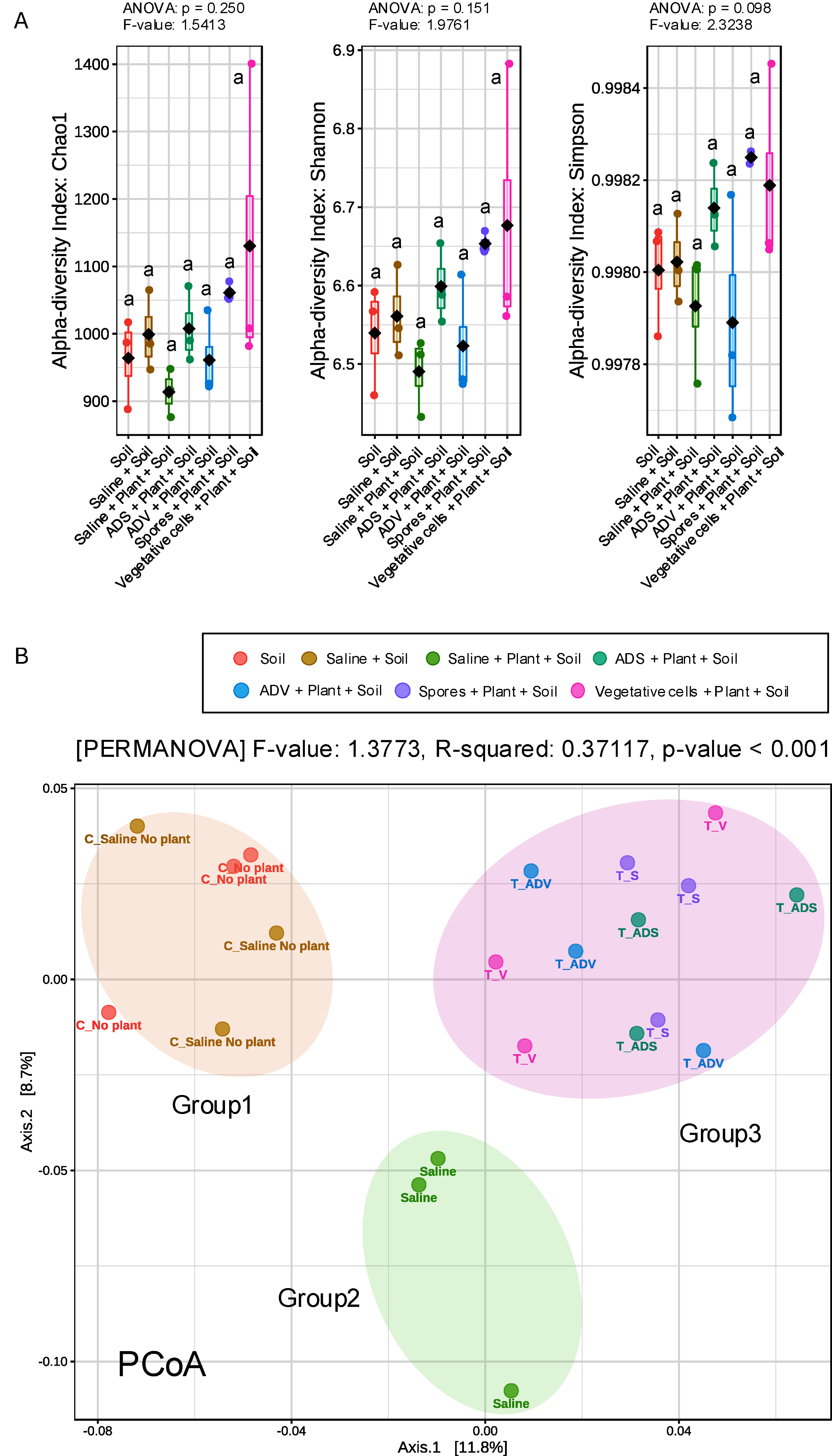
Figure 3. Influences of treatments on the bacterial microbiota in soil. A, a measurement of alpha diversity. The alpha diversity analysis utilized the Chao1, Shannon, and Simpson indices, with three replicates. B, a measurement of beta diversity. Principal coordinate analysis (PCoA) was conducted using Bray–Curtis distance metrics for taxonomic data (*p*<0.001). Permutational multivariate analysis of variance (PERMANOVA) was also performed.

Bacterial alpha diversity, indicating their richness, and beta diversity, indicating their composition, are displayed in the Figure 3. Regardless of treatment, alpha diversities, i.e., Chao1, Shannon, and Simpson, did not show significance in the analysis of variance (ANOVA) (Figure 3A). This suggests that bacterial application, regardless of viability, does not dramatically alter bacterial richness in soil. Meanwhile, beta diversity plotted on the principal coordinate analysis (PCoA) was separated into 3 groups with statistical significance. Group 1 comprised 2 treatments, original soil and incubated soil (Saline+Soil), while the incubated soil with plants (Saline+Plant+ Soil) was clustered into group 2 (Figure 3B). All bacterial treatments, including the autoclaved dead vegetative cells and spores, did not separate the group. Hence, beta diversity varied among treatments, forming 3 distinct groups: (1) the soil bacterial community without plants, (2) the soil bacterial community with plants, and (3) the soil bacterial community with plants influenced by external inoculation. The formation of these three groups may be attributed to distinct selective pressures acting on the soil microbiome. The first group (soil without plants) represents the native microbial community structure, which remains largely unchanged due to the absence of plant-microbe interactions. The second group (soil with plants) likely reflects the selective influence of *Setaria viridis*, which can shape the rhizosphere microbiome by exuding root exudates that promote certain microbial taxa. The third group (soil with plants and bacterial inoculation) suggests an additional modification of the microbial community driven by the introduced bacteria, whether through direct competition, cross-feeding interactions, or indirect effects such as alterations in nutrient availability or secondary metabolite production. However, there was no extreme modification on top 35 taxa abundance, suggesting the overall bacterial community composition remained relatively stable across treatments (Supplementary Figure S3A).

**Figure 4:**
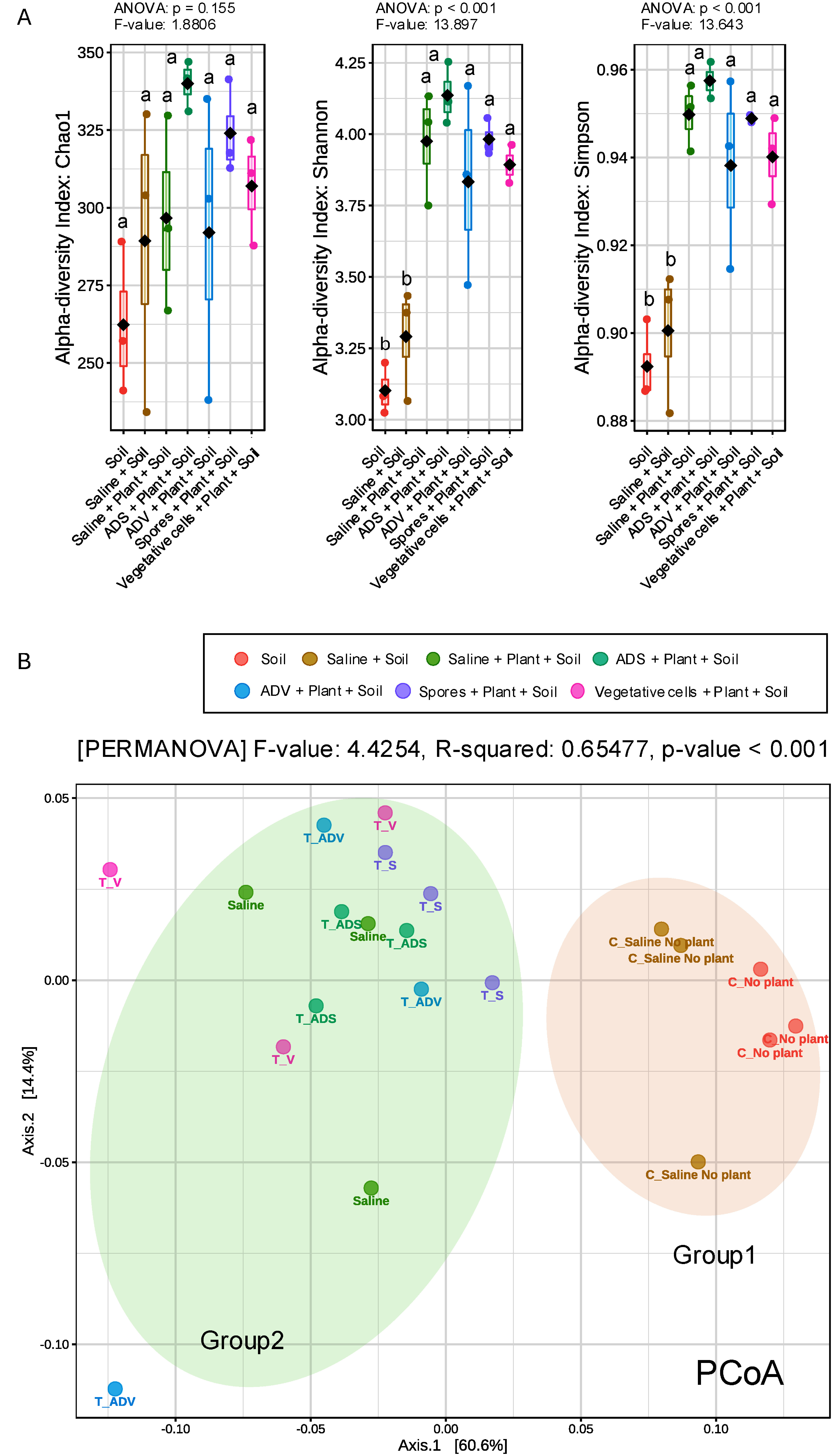
Figure 4. Influences of treatments on the fungal microbiota in soil. A, a measurement of alpha diversity. The alpha diversity analysis utilized the Chao1, Shannon, and Simpson indices, with three replicates. Statistical significances among treatments were determined using post-hoc Tukey’s test (*p*<0.05) based on the ANOVA results, with different letters indicating significant differences. B, a measurement of beta diversity. Principal coordinate analysis (PCoA) was conducted using Bray–Curtis distance metrics for taxonomic data (*p*<0.001). Permutational multivariate analysis of variance (PERMANOVA) was also performed.

Fungal alpha and beta diversities are indicated in the Figure 4. Two of the alpha diversity indices, Shannon and Simpson, showed significance differences between treatments and the post-hoc multiple comparisons calculated using the Tukey’s test indicated the planting of *Setaria* impacted fungal communities regardless of inoculation treatment (Figure 4A). The beta diversity separated into 2 distinct groups mirroring the alpha diversity; i.e., (1) the soil fungal community without plants and the (2) soil fungal community with plants unaffected by bacterial inoculation (Figure 4B). The taxa abundance between the two groups also differed in *Mortierella* spp. significantly suppressed by planting *Setaria* even though this genus is reported as an endophytic PGPF (Supplementary Figure S3B) (Johnson et al. 2019; Ozimek and Hanaka 2021).

**Figure 5:**
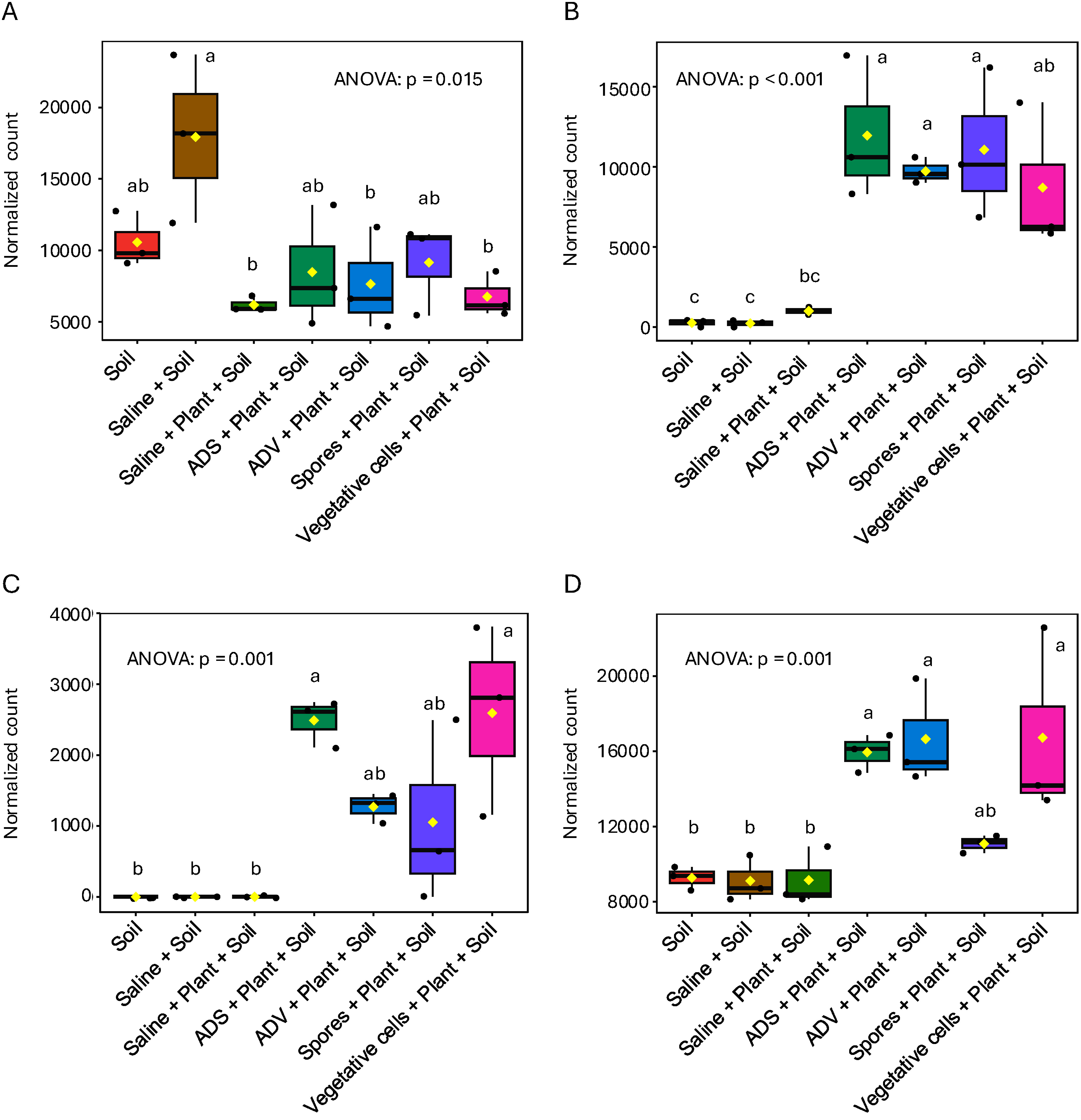
Figure 5. Normalized count data of representative genera as a function of bacterial treatments. A, *Bacillus*. B, *Bdellovibrio*. C, *Bacteriovorax*. D, *Pseudomonas*. Statistical significances among treatments were determined using post-hoc Tukey’s test (*p*<0.05) based on the ANOVA results, with different letters indicating significant differences (*n*=3).

The top 15 features enriched in linear discriminant analysis effect size (LEfSe) and their heatmap are displayed in Supplementary Figure S4. A notable pattern dividing two groups with or without bacterial inoculations was obtained where *Pseudomonas* spp., *Bdellovibrio* spp., and *Bacteriovorax* spp. were enriched. Interestingly, one of *Bacillus* spp. features, ASV_057, was enriched but showed a different pattern with higher numbers in soil without plants and lower numbers in soil with plants. The normalized count data of those representative genera are shown in Figure 5. The abundance of *Bacillus* in bacterial treatments was reduced even though viable spore and viable vegetative cell treatments have the capability to proliferate their population and all bacterial treatments contain *Bacillus* 16S rDNA (Figure 5A). This result emphasizes the robustness of the microbial community and suggests that the external bacterial biopolymers, such as DNA, may have been decomposed by the microbial community (Nielsen et al. 2007).

The abundances of *Bdellovibrio* spp. and *Bacteriovorax* spp. were significantly increased upon bacterial inoculation regardless of use of dead or live cells (Figure 5B, C). *Bdellovibrio* and *Bacteriovorax* are gram-negative bacteria well-known as predators which consume bacterial biopolymers (Chen et al., 2012; Lovering and Sockett 2021). This indicates their propagation was facilitated by provided inoculants. Both these species are known to prey on gram-negative bacteria, not gram-positive bacteria, suggesting those predators increased their populations by consuming the gram-negative bacteria that proliferated as a result of bacterial treatments. In addition, members of the *Pseudomonas* genus, some of which are well known as PGPR, were also enriched in abundance, which might contribute to plant growth promotion of *Setaria* (Figure 5D) (Qessaoui et al. 2019). However, there were no enriched patterns separating spores and vegetative cells on bacterial LEfSe, and none of the clear traits upon inoculation were enriched in the fungal analysis.

In conclusion, endospores contained higher levels of carbon, calcium, and manganese, which are linked to their protective characteristics. Interestingly, all treatments, including viable and dead states of endospores and vegetative cells, promoted plant growth on natural soil, suggesting that growth-promoting effects could, at least in part, be mediated by microbial communities rather than viable bacterial activity or residues. Microbial community analysis revealed that bacterial alpha diversity between planting or treatments was not changed but beta diversity varied significantly, while fungal communities showed increased alpha diversity with *Setaria* planting but were not significantly influenced by bacterial treatments. These results highlight that bacterial inoculation alters the soil microbial community structure. However, while certain bacterial genera such as *Bdellovibrio*, *Bacteriovorax*, and *Pseudomonas* were enriched, further studies are needed to establish a direct causal link between these microbial changes and plant growth promotion. This study underscores the potential of even bacterial dead cells in sustainable agriculture to influence microbial ecosystems and promote plant development.

## Abbreviations

PGPF: plant growth promoting fungi
PGPR: plant growth promoting rhizobacteria

## Description of supplementary files

Supplementary Table S1. Primers for the library preparation.

Supplementary Figure S1. Plant growth at 14 days after transplanting.

Supplementary Figure S2. Rarefaction curves.

Supplementary Figure S3. Relative abundances of the genera on soil.

Supplementary Figure S4. The top 15 features enriched in the linear discriminant analysis effect size (LEfSe) with a significance (*p*<0.05) and their heatmap.
