# Supplementary material for: Comparative analysis of Bacillus pumilus TUAT1 endospores and vegetative cells: Implications for plant growth promotion and soil microbiome modulation
Source: Plant Biotechnol (Tokyo). 2025 Sep 25;42(3):327–34. doi: 10.5511/plantbiotechnology.25.0328a (PMC12573575; doi:10.5511/plantbiotechnology.25.0328a)
Supplement: Supplementary Data [file plantbiotechnology-42-3-25.0328a_s001.pdf]

Supplementary Files

Comparative analysis of *Bacillus pumilus* TUAT1 endospores and vegetative cells: Implications for plant growth promotion and soil microbiome modulation

Shin-ichiro Agake, Jean Louise Cocson Damo, Hiroki Rai, Gary Stacey, Michiko Yasuda, Naoko Ohkama-Ohtsu

Supplementary Table S1

Supplementary Table S1. Primers for the library preparation.

Primers	Sequences
1st_PCR_V3V4f_MIX（341f)：	ACACTCTTTCCCTACACGACGCTCTTCCGATCT-NNNNN–CCTACGGGNGGCWGCAG
1st_PCR_V3V4r_MIX（805r)：	GTGACTGGAGTTCAGACGTGTGCTCTTCCGATCT-NNNNN–GACTACHVGGGTATCTAATCC
1st_ITS1-F_KYO1：	ACACTCTTTCCCTACACGACGCTCTTCCGATCT-NNNNN–CTHGGTCATTTAGAGGAASTAA
1st_ITS2_KYO2：	GTGACTGGAGTTCAGACGTGTGCTCTTCCGATCT-NNNNN–TTYRCTRCGTTCTTCATC
2nd Forward primer：	AATGATACGGCGACCACCGAGATCTACAC-Index*-ACACTCTTTCCCTACACGACGC
2nd Reverse primer：	CAAGCAGAAGACGGCATACGAGAT-Index*-GTGACTGGAGTTCAGACGTGTG

Index* is the sequence for sample identification.

Supplementary Figure S1

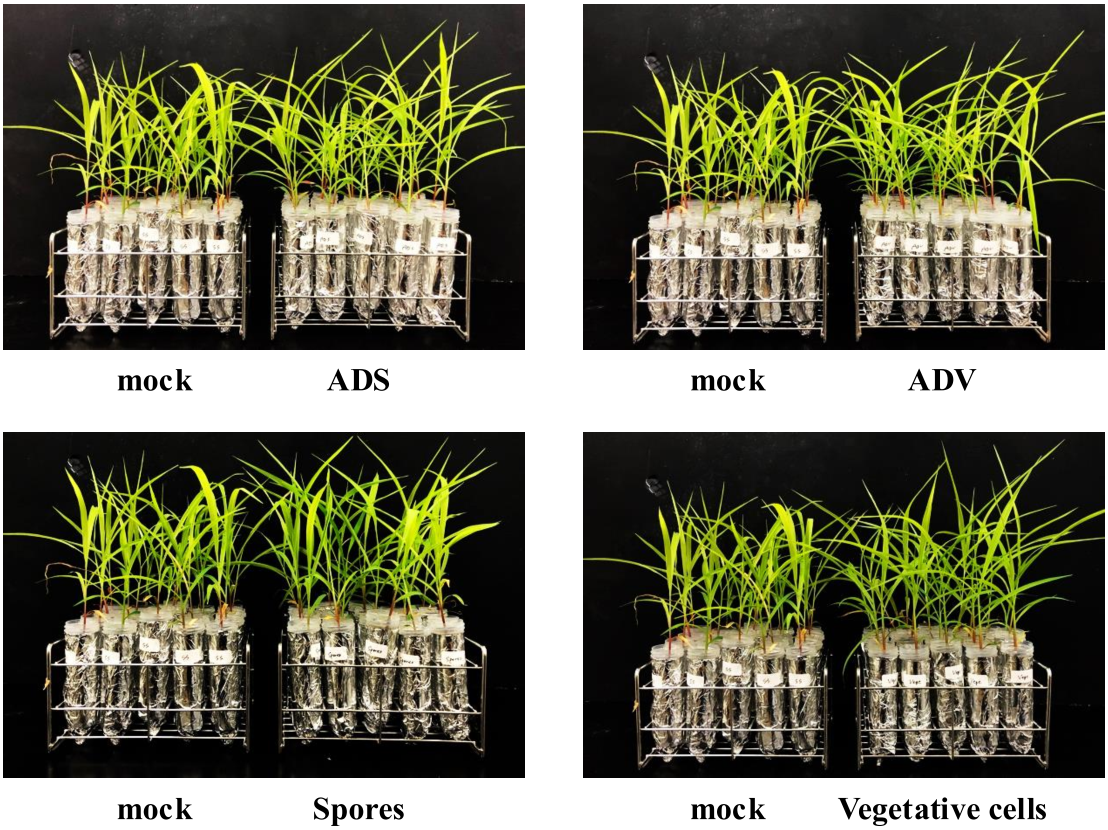

Supplementary Figure S1. Plant growth at 14 days after transplanting.

Supplementary Figure S2

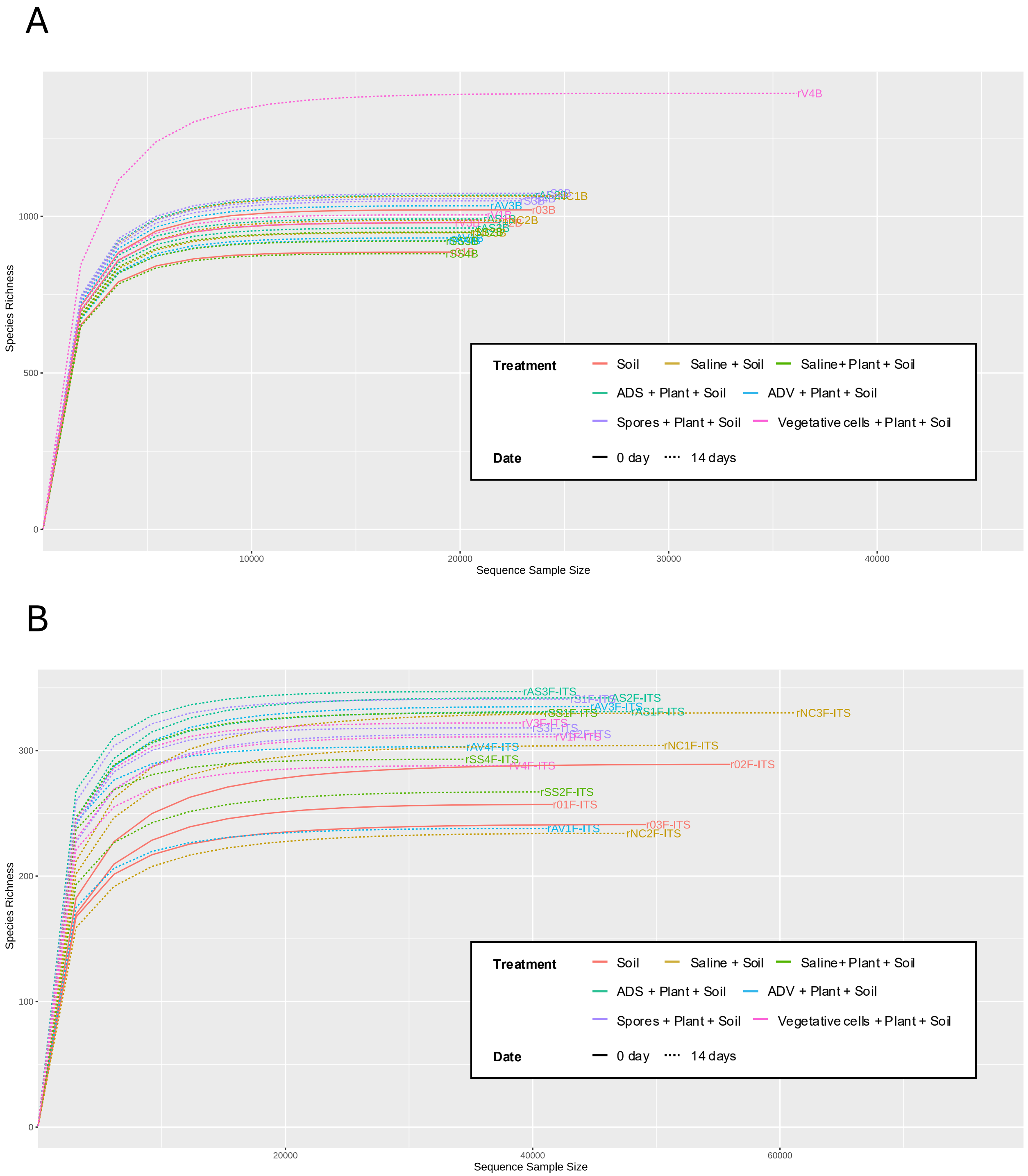

Supplementary Figure S2. Rarefaction curves. A, Bacterial microbiome. B, Fungal microbiome.

Supplementary Figure S3

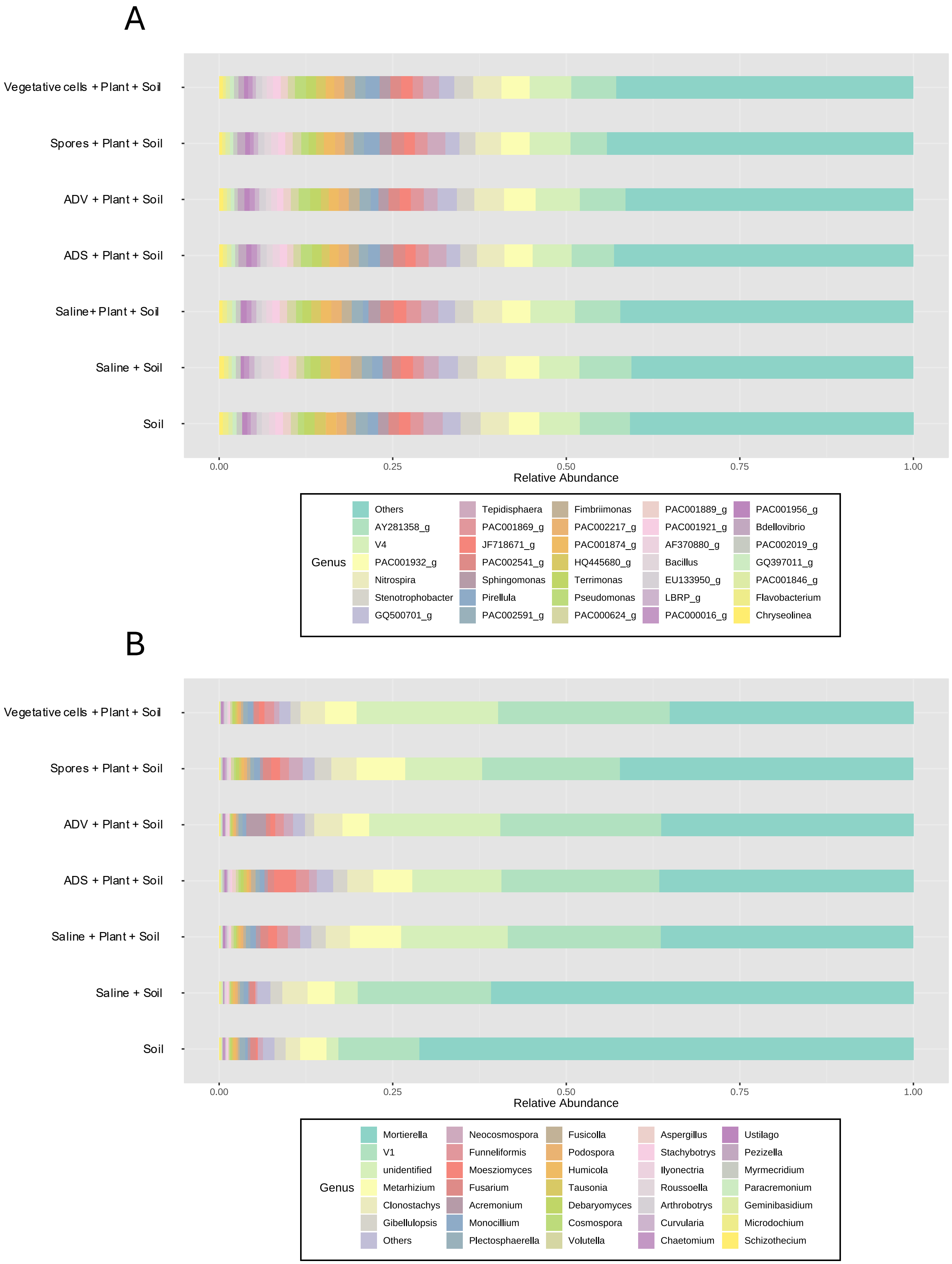

Supplementary Figure S3. Relative abundances of the genera on soil. A, microbial taxa abundance with top 35 genera. B, fungal taxa abundance with top 35 genera.

Supplementary Figure S4

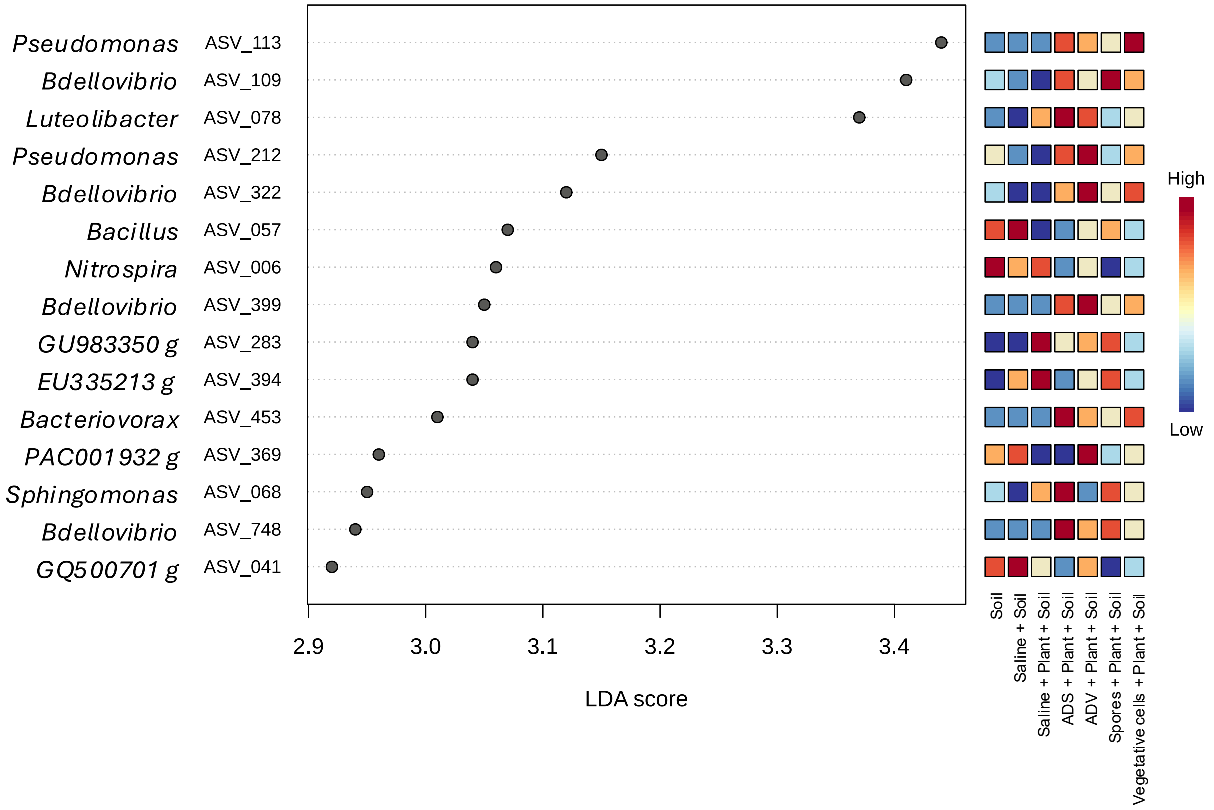

Supplementary Figure S4. The top 15 features enriched in the linear discriminant analysis effect size (LEfSe) with a significance ($p<0.05$) and their heatmap. The heatmap represents the abundance of each feature among treatments. Blue color indicates lower normalized counts and red indicates higher normalized counts.
